# Exploring the change of coastal saline agroecosystem diversity, composition and predicted function of soil microbial community mediated by soybean and corn intercropping

**DOI:** 10.3389/fpls.2025.1427196

**Published:** 2025-06-26

**Authors:** Witness Joseph Nyimbo, Nyumah Fallah, Arcel Mulowayi Mutombo, Hailong Xu, Qin Bin, Janeth Samwel Makungu, Wenxiong Lin

**Affiliations:** ^1^ College of Life Sciences, Fujian Agricultural and Forestry University, Fuzhou, China; ^2^ Agricultural Ecology Research Institute, Fujian Agriculture and Forestry University, Fuzhou, China; ^3^ College of Agriculture, Fujian Agricultural and Forestry University, Fuzhou, China; ^4^ College of Mechanical and Electrical Engineering, Fujian Agriculture and Forestry University, Fuzhou, China

**Keywords:** coastal saline agroecosystem, soybean-corn intercropping, soil microbial community, microbial functions, climate change

## Abstract

**Introduction:**

Soil microbial community is the key determinant of coastal agroecosystem soil health. However, the response of soil microbial community and its anticipated functions to soybean and corn intercropping in coastal saline agroecosystems is not well understood.

**Method:**

Soybean and corn intercropping was done in Putian city of Fujian province. After harvest, soil total carbon (TC), total phosphorus (TP), total nitrogen (TN), total organic carbon (TOC), soil organic matter (SOM), salinity content and elemental ratios of C: N, C: P and N: P were examined. High-throughput sequencing was performed to investigate the community composition and diversity of rhizospheric bacterial and fungal communities as influenced by monoculture soybean (MS) and corn (MC), first (FP) and second (SP) intercropping pattern. LEfSe cladogram was generated to identify potential microbial markers and metagenome was annotated with the metabolic cycles and pathways in the KEGG database to predict the microbial function. The co-occurrence and RDA analysis assessed the correlation between microbes and soil microbes with soil chemical parameters.

**Results and discussion:**

The intercropping patterns FP and SP significantly influenced soil TC, TP, TN, SOM, EC, pH and salinity content. The C: N, C: P, and N: P ratios were influenced by C, N, and P concentrations. Our investigation found that Chao1 was significantly higher in intercropping patterns than in monoculture patterns. Nevertheless, the Shannon index was substantially higher in monoculture than in intercropping patterns FP and SP indicating reduced bacterial and fungi diversity measured by species richness and evenness. The Non-Metric multidimensional scaling (NDMS) diversity showed that all samples were significantly clustered into four major groups, according to the bacteria and fungi communities of origin. Further statistical analysis revealed that cropping patterns strongly affected microbial communities. Furthermore, Proteobacteria, Actinobacteria, Acidobacteria, and Chloroflexi were enriched bacterial phyla in the rhizosphere of MS, MC, FP, and SP. Ascomycota, Mortierellomycota, and Basidiomycota were the most enriched fungi phyla in each intercropping pattern. These phyla were identified as sensitive biomarkers for soil nutrient circulation, ecosystem bioremediation and chemical degradation.

**Conclusion:**

This study increases our understanding of soybean and corn intercropping in coastal saline agroecosystems microbiomes

## 1 Introduction

Coastal agroecosystem soil is a complex ecosystem hosting bacteria, fungi, and other soil microorganisms (Jacoby et al., 2017). Soil microorganisms encompass a significant amount of soil ecosystem’s genetic diversity (Bashir et al., 2016). There is substantial evidence suggesting soil microbial community being a crucial predictor of soil quality and ecosystem functions, such as climate regulation and bioremediation (Chaer and Goedert, 2013; Jiang et al., 2022). Soil microbes regulate global climate by sequestering carbon dioxide (CO_2_) from the atmosphere through carbon fixation and storing it in the form of organic matter (Dong et al., 2022). In nitrogen (N) cycling, the fixation of atmospheric N into plant-available ammonium (NH_4_) is performed by N fixers (Tibbett et al., 2020). While nitrification of ammonia and denitrification of nitrate into N oxide and N gas is exclusively performed by diazotrophs such as rhizobia, nitrifiers such as nitrobacter and denitrifiers such as pseudomonas (Li et al., 2021). Furthermore, these microbes are significant in soil biological activities such as decomposition of organic matter, plant diversity, and productivity (Zhou et al., 2020).

However, over-exploitation and climate change events can significantly impair the health and function of coastal agroecosystems (Leonardo et al., 2020). The slight change in climate in coastal agroecosystems can cause significant and rapid changes in sea levels, exceeding the capacity of coastal ecosystems to adapt (Tu Nguyen et al., 2019). The climate change-related abiotic stresses, such as drought, an increase in salinity, heat waves and floods, are the primary cause of ecosystem fluctuations in the structure and functioning of soil microbes (Bardgett et al., 2020). The change in species diversity and composition can alter the ecosystem functions and processes associated with productivity and ecosystem health (Wagg et al., 2021; Sahab et al., 2021).

To solve the coastal agroecosystem challenges, intercropping of soybean and corn was observed to be among the practical approaches to lessen the negative impacts caused by coastal agroecosystem changes (Iqbal et al., 2019). Soybean and corn intercropping can shape the functions of soil microbes in coastal agroecosystems (Bastida et al., 2021). For example, soybeans improve soil nutrients by fixing N from N dioxide (N_2_) and carbon sequestration (Stagnari et al., 2017). Also, soybeans facilitate the conversion of unavailable P into mobilized P, benefiting themselves and the neighboring plant (Duchene et al., 2020). On the other hand, corn is one of the most important crops in the world (Li et al., 2019). Corn plays an integral economic and nutritional role and is also an exhaustive crop that acquires mineral N from the soil (Acevedo-siaca and Goldsmith, 2020). The intercropping of soybeans and corn not only influences soil essential nutrient circulation but also increases soil microbial biodiversity and composition of coastal agroecosystem soil, which are important indicators of soil agroecosystem health (Maitra et al., 2021).

Soil chemical properties affect microbial community diversity, composition, and function of ecosystems (Dang et al., 2020). For example, soil TN, TP, TC, soil organic matter (SOM) and pH affect soil microbial community composition and function (Song et al., 2020). Study on soil C, N and P suggest that there is a relationship between soil nutrients and their corresponding soil elemental ratios (C:N, C:P and N:P) which reflects organic matter decomposition, nutrient retention, and nutrient circulations (Liu et al., 2016). Hence, in studying the change of diversity, composition and predicted function of soil microbial community it is important to include elemental ratios since their response to coastal agroecosystems has not been extensively investigated (Wu et al., 2015). This work focuses on (i) Analyzing the potential effects of soybean and corn in intercropping on the soil microbial community of Putian agroecosystem soil, (ii) Predicting the soil microbial functions mediated by soybean and corn intercropping in agroecosystem and (iii) To establish the relationship between the intercropping patterns, soil chemical factors and significant composition within microbial communities in Putian coastal agroecosystem soil microbiome.

## 2 Methods and experiments

### 2.1 Site description

Soil samples were collected from Xiuyu District, Putian City, Fujian Province, China (longitude 118° 72 E latitude 27° 43N) at an altitude of 150 m above sea level. The mean annual rainfall was 1,650 mm, with a mean temperature of 19°C, of which more than 80% of rainfall is concentrated from April to July each year. The soil type was sandy loam with pH of 7.6, EC 4.6 dSm^-^1, TP of 0.716 g/Kg, TN of 0.78 g/Kg, TC of 3.97 g/Kg, and SOM of 9.02 g/kg. The salinity content of the rhizospheric soil was above 4 g/kg, which, according to Xu et al. (2020), was categorized as severe saline soil.

### 2.2 Field experiment

**Figure 1:**
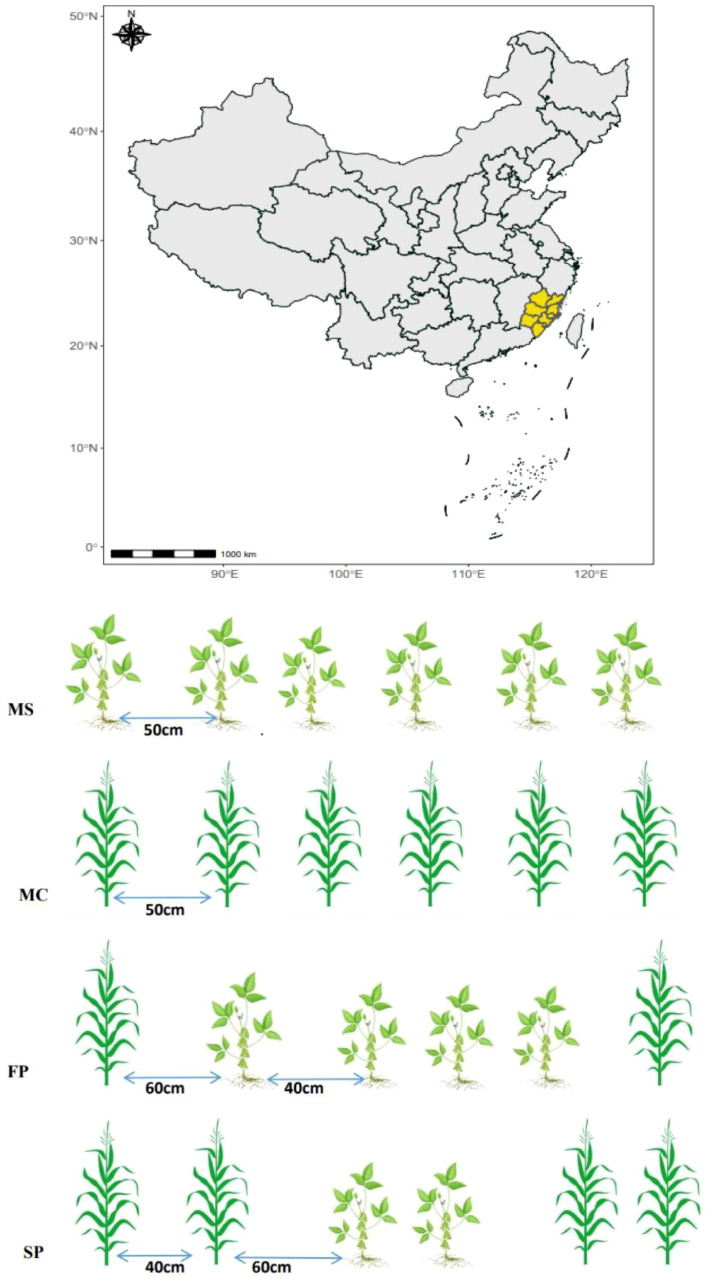
Showing the map of the experimental site and intercropping patterns.

This experiment consisted of monoculture soybean (MS), monoculture corn (MC), a first pattern (FP) and a second pattern (SP). The plots were separated into 12 sub-plots; each treatment had three replicates. The soybean variety used was Gui-105, and the corn used was Caignuo 8, purchased from Wannong High-Tech (Group) Co., Ltd. The Base fertilizer used was urea, and the compound fertilizer was N-P_2_O_5_-K_2_O, which was applied separately, consisting of 14.5 and 55 g m^–2^, respectively. After two days, monoculture corn and soybean were sown directly into the soil; the distance between one row to another was 50 and 40 cm between one plant and another in MC and MS. In the intercropping FP and SP plots, the distance between corn and soybean was 60 cm, while the distance between soybean and soybean was 40 cm. The buffer zone of 60 cm from the edge of the plot in the corn and soybean intercropping plots was maintained to avoid the influence of adjacent plots (**Figure 1**). All experimental plots were managed using conventional field management practices, such as weeding and insecticide application.

### 2.3 Sample collection and processing

After harvest, 3 rhizosphere soil samples were collected and bulked together to form one sample per plot using the S-sampling method. The samples were immediately stored in sterile plastic bags, placed in iceboxes, and taken to the laboratory. Then, all the samples were sieved through a 2-mm mesh and thoroughly homogenized to be further divided into two parts: one was air-dried for soil chemical analysis, while the rest were stored at ^−^80°C, waiting for DNA extraction.

### 2.4 Determination of chemical properties and elemental ratios analysis

A potassium dichromate bulk density method was used to determine SOM (Bai et al., 2017). Kjeldahl’s method was used to measure soil TN (Nutrien and Pengurusan, 2019). Soil TP was determined by the HClO_4_–H_2_SO_4_ method (Ma et al., 2023). Soil TOC and TC were determined by elemental analyzer (Djukic et al., 2013). The soil C: N, C:P, and P: N element ratios were calculated according to (Wu et al., 2015).

### 2.5 Coastal salinity content

Soil pH was measured using the micro-electrode method (Yang et al., 2020). Soil salinity content was analyzed using the conversion equation of salinity and EC [Equation 1] (Dong et al., 2022).


(1) Salinity=(EC/1000−(0.0182)/0.39

Where the unit of EC was µS/cm, and the salinity content unit was in g/Kg.

### 2.6 Microbial composition and diversity assessment

The soil genomic DNA was extracted using the E.Z.N.A. Soil DNA Kit (Omega Bio-tek Inc., USA). The concentration and quality of genomic DNA was measured by NanoDrop 2000 spectrophotometer (Thermo Scientific Inc., USA). The V4 region of the 16S rRNA sequence was amplified by universal primers 338F (5’ACTCCTACGGAGGCAGCAG-3’) and 806R (5’GGACTACNNGGGTATCTAAT-3’) (Dang et al., 2020). According to Smith and Peay (2014), the fungal ITS region was amplified by ITS1F (5’CTTGGTCATTTAGAGGAAGTAA-33’), an ITS1 region target. PCR was conducted on a Mastercycler Gradient (Eppendorf, Germany) using 25 μL reaction volumes with 12.5 μL 2× Taq PCR MasterMix (Vazyme Biotech Co., Ltd, China), 3 μL BSA (2 ng/μl), 1 μL forward primer (5 μM), 1 μL reverse primer (5 μM), 2 μL template DNA and 5.5 μL ddH2O. The cycling conditions were 95°C for 5 minutes, followed by 28 cycles of 45s at 95°C, 50s at 55°C, and 45s at 72°C. We purified the PCR products using AMPure XP (Beckman Coulter Inc., USA). We employed the NEB Next Ultra II DNA Library Prep Kit from New England Biolabs, Inc. in the United States to produce the DNA libraries. We employed three tools: the Nanodrop 2000, the Agilent 2100 Bioanalyzer, and the ABI StepOnePlus Real-Time PCR system. The Deep Sequencing was conducted at Beijing Allwegene Technology Co., Ltd. using the Illumina Miseq/Novaseq platform (Illumina, Inc., USA). Illumina Analysis Pipeline v. 2.6 (Illumina, Inc., USA) was utilized for image analysis, base calling, and error evaluation (Zhang et al., 2014). Sequence data from this project are deposited in the NCBI Short Read Archive (accession no. PRJNA1224044).

### 2.7 Data processing and bioinformatics analysis

The raw data for the 16S rRNA and ITS genes were separated into samples based on the barcode sequence using Pear (v0.9.6) software (Zhang et al., 2014). The sequences contained ambiguous bases N were removed and the parts with low-quality scores (≤ 20) were cut off from the sequences. During splicing, the minimum overlap setting was 10bp and the p-value was 0.0001. After splicing, Vsearch (v2.7.1) software was used to remove the 16S sequences with lengths less than 230 bp and the chimeric sequence by the Uchime method, according to the Gold Database (Rognes et al., 2016). In contrast, ITS2 was 230 bp and removed the chimeric sequence by Uchime method, according to the Unite Database (Ramírez-Guzmán et al., 2004). The qualified sequences were clustered into taxonomic units (OTUs) using the Vsearch software’s Uparse algorithm and the similarity threshold was set at 97% (Gao et al., 2016). The BLAST tool was used to classify all OTU sequences into different taxonomic groups according to the Silva138 Database for the 16S rRNA gene and the Unite8.2 Database for the ITS gene (Rognes et al., 2016).

The Chao1 and Shannon indices were used to visualize the richness and diversity of the microbial communities (Xu et al., 2020). These indices were computed for each sample using the amplicon sequence variants (ASVs) database, limited to 20,349 sequences in QIIME2 (v1.8.0). The nonmetric multidimensional scaling (NMDS) was used to describe variation in fungal and bacterial community structures (Mang et al., 2021). The Bray-Curtis was used to visualize the differentiation among samples. Adonis R function for multivariate analysis was used to compare microbial communities with a significance level of P < 0.05. The results were visualized using R (v3.6.0) software.

LEfSe analyzed the potential biomarkers taxa. The linear discriminant analyses (LDA) log score >3.7 were considered for interpretation, and Python (v2.7) software was used for LEfSe (Segata et al., 2011) analysis. Vegan R package and PICRUSt2 were used to compare the similarity of soil bacteria and fungi and their functional profiles based on the P-values and goodness of fit (m2). Also, the co-occurrence network construction and analysis were done by igraph, and Hmisc packages in R.

### 2.8 Statistical analysis

The influence of cropping patterns on soil chemical properties was performed by One way ANOVA using graph-pad prism (version 6.01). The least significant difference (LSD) test was used to compare the mean between samples at p<0.05 significant level and results were plotted by graph-pad prism. Meanwhile, Spearman’s correlation analysis of soil microbial composition and abundances to environmental factors and visualized by R with a vegan package (version 3.4.2).

## 3 Results

### 3.1 Effects of soybean-corn intercropping on soil chemical properties, element ratios and coastal salinity content

**Figure 2:**
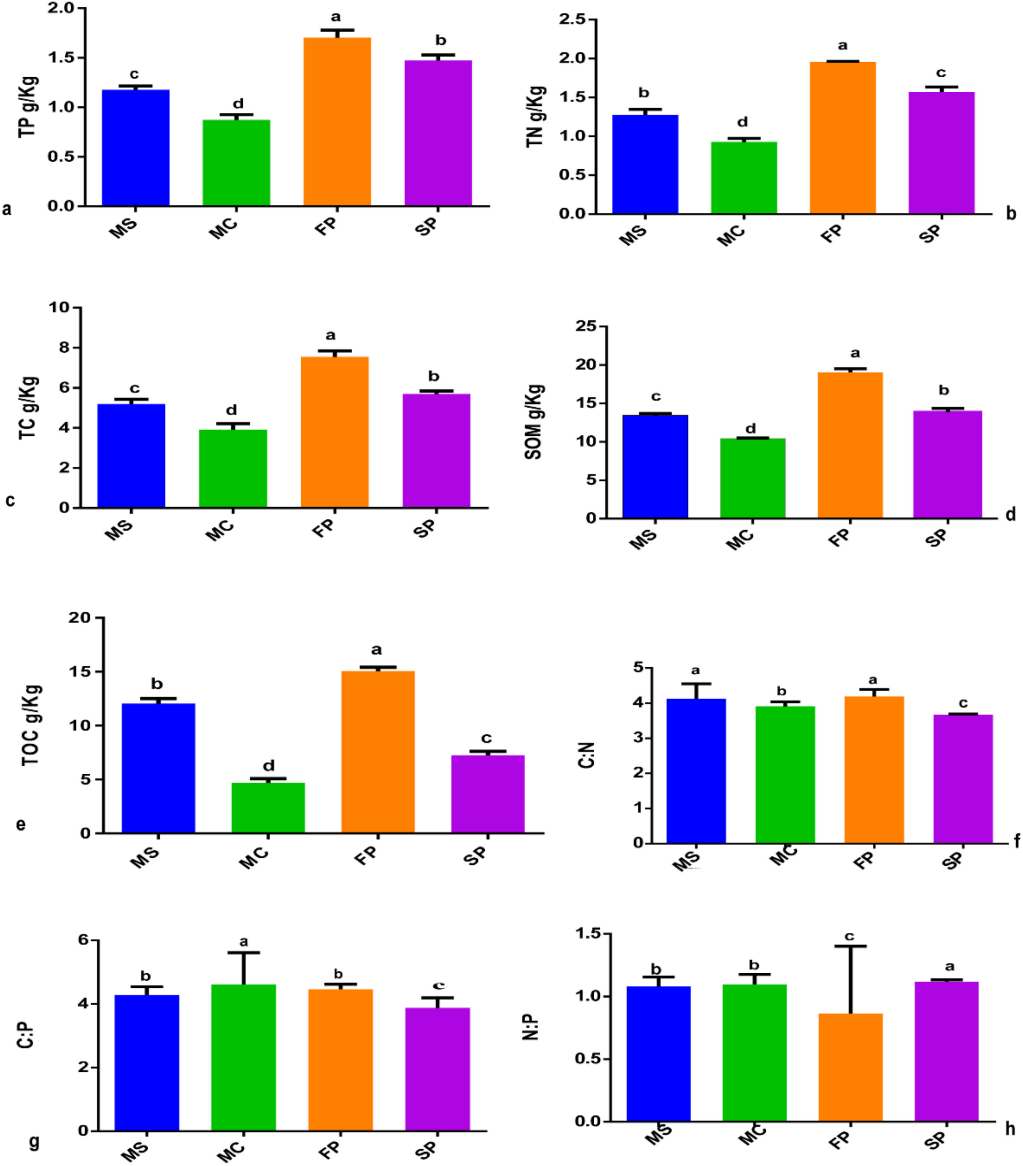
Soil chemical properties showing the effects of intercropping in four cropping patterns **(a)** TP **(b)** TN **(c)** TC **(d)** SOM **(e)** TOC **(f)** C:N **(g)** C:P and **(h)** N:P. MS; monoculture soybean, MC; monoculture corn, FP; First intercropping pattern and SP; second intercropping patterns. The bars represent the standard errors of mean of intercropping patterns (n = 3). Lowercase letters mean difference between different cropping patterns (*P* < 0.05).

**Figure 3:**
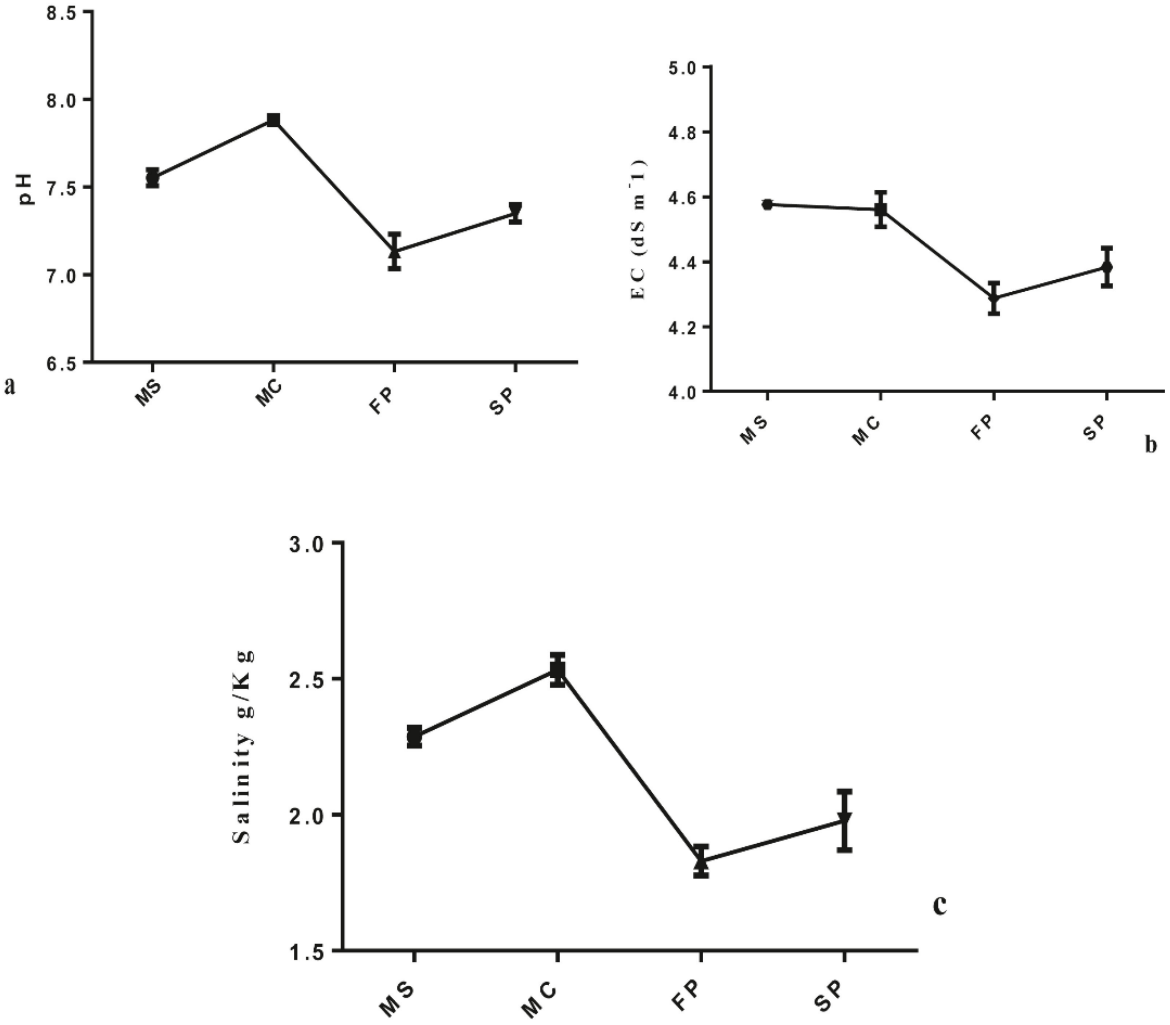
Four cropping patterns **(a)** pH **(b)** EC and **(c)** salinity content. MS; monoculture soybean, MC; monoculture corn, FP; First intercropping pattern and SP; second intercropping patterns.

The study revealed that cropping patterns significantly influenced the concentration of soil chemical properties. Results showed that soil TC, TP, TN and SOM concentration was significantly higher in FP followed by MS > SP> MC at (P< 0.05). Meanwhile, TOC was marked to be significantly higher in FP, followed by MS>SP>MC (P< 0.05). Also, results indicated that the C: N, C: P, and P: N ratios were significantly influenced by cropping patterns. The C: N and C: P ratios showed no significant difference between monoculture MS and intercropping FP. However, significant differences were observed in MC, FP, and SP (**Figures 2f, g**). In MC and SP N: P ratio was significantly higher followed by MS and FP (**Figure 2h**). Result pointed out that the salinity content, EC, and pH were significantly reduced in intercropping patterns FP, and SP at (P < 0.05). The salinity content, EC, and pH of rhizospheric soil were reduced considerably from FP>SP> MC >MS at P<0.05. Compared to all intercropping patterns, the salinity content, EC, and pH o were higher, in monoculture MC followed by MS, at P < 0.05 (**Figure 3**).

### 3.2 Effects of soybean-corn intercropping on soil microbial community diversity

**Figure 4:**
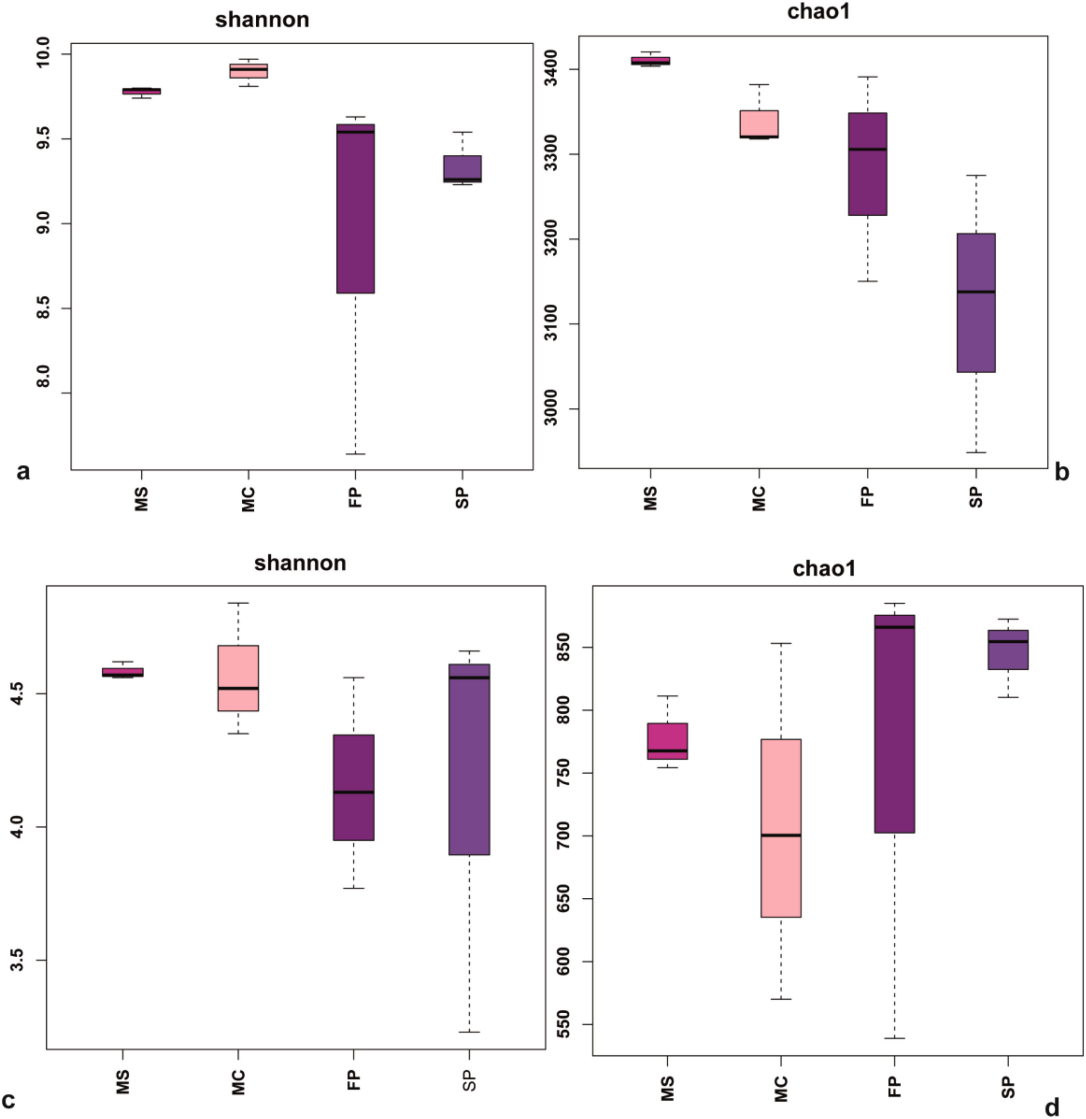
Box plots showing **(a)** Shannon indices **(b)** Chao1 based on bacteria and **(c)** Shannon diversity indices **(d)** Chao1 richness based fungi communities in the soil samples. MS; monoculture soybean, MC; monoculture corn, FP; First intercropping pattern and SP; second intercropping patterns.

The 503,330 raw sequences and an average of 41,944 sequences per sample were obtained. After quality control, sample normalization, and filtration, samples were clustered into 6,745 unique sequences, the sample coverage was >93%. The rarefaction curve of each sample reached a saturation plateau, showing that the obtained sequencing depth reflected the soil bacteria and fungi composition (**Supplementary Figure 1**). Results indicated that the intercropping patterns affected the Shannon–Wiener index of the microbial community. The Shannon-Wiener diversity indexes followed the MC > MS >FP >SP trend. Also, it was noted that the bacterial Chao1 and Shannon indices were higher in the MS and MC compared with the FP and SP. However, the diversity indices in fungi Chao1, are higher in the FP and SP patterns than in monoculture patterns. Nevertheless, the Shannon index was higher in the MC and MS than in FP and SP, indicating that the species richness with evenness reduced the bacterial and fungal diversity measured by the species richness (**Figure 4**).

The non-multidimensional scaling (NDMS) of rhizospheric soil was performed to compare the bacterial and fungal community diversity of MS, MC, FP, and SP. We also found that samples from the same cropping pattern clustered tightly. Results pointed that the fungal and bacterial community structure was strongly affected by soybean and corn intercropping patterns. (**Supplementary Figure 2**).

Adonis R function was used to analyze the soil microbial compositions under various treatments. The results showed significant differences between various cropping patterns. The PERMANOVA results showed that the bacteria and fungi communities differed significantly (R^2^ = 0.185, p = 0.001) and (R^2^ = 183, p=0.001), respectively. Intercropping patterns significantly influence soil microbial communities (**Tables 1**, **2**).

**Table 1:** Adonis multivariate analysis of estimating the beta-diversity of bacteria under four cropping patterns.

Cropping Pattern	Df	Sum Of Sqs	R2	F	Pr(>F)
Group	3	0.556	0.185413	4.21121	<0.001*
Residual	8	0.352	0.0440284		
Total	11	0.908			

Statistical analysis is expressed by P-value, and P< 0.001 indicates that the statistics are significant. Df, Degrees of freedom; Sum Of Sqs, sum of Square; R2, R square; F, F-value by permutation; Pr, >F, P value.

**Table 2:** Adonis multivariate analysis of estimating the beta-diversity of fungi under four cropping patterns.

Cropping Pattern	Df	Sum Of Sqs	R2	F	Pr(>F)
Group	3	0.825141	0.275047	4.22622	<0.001*
Residual	8	0.520649	0.0650811		
Total	11	1.34579			

Statistical analysis is expressed by P-value, and P< 0.001 indicates that the statistics are significant. Df, Degrees of freedom; Sum Of Sqs, sum of Square; R2, R square; F, F-value by permutation; Pr, >F, P value.

### 3.3 Effect of soybean-corn intercropping on the composition of soil bacteria and fungi community

**Figure 5:**
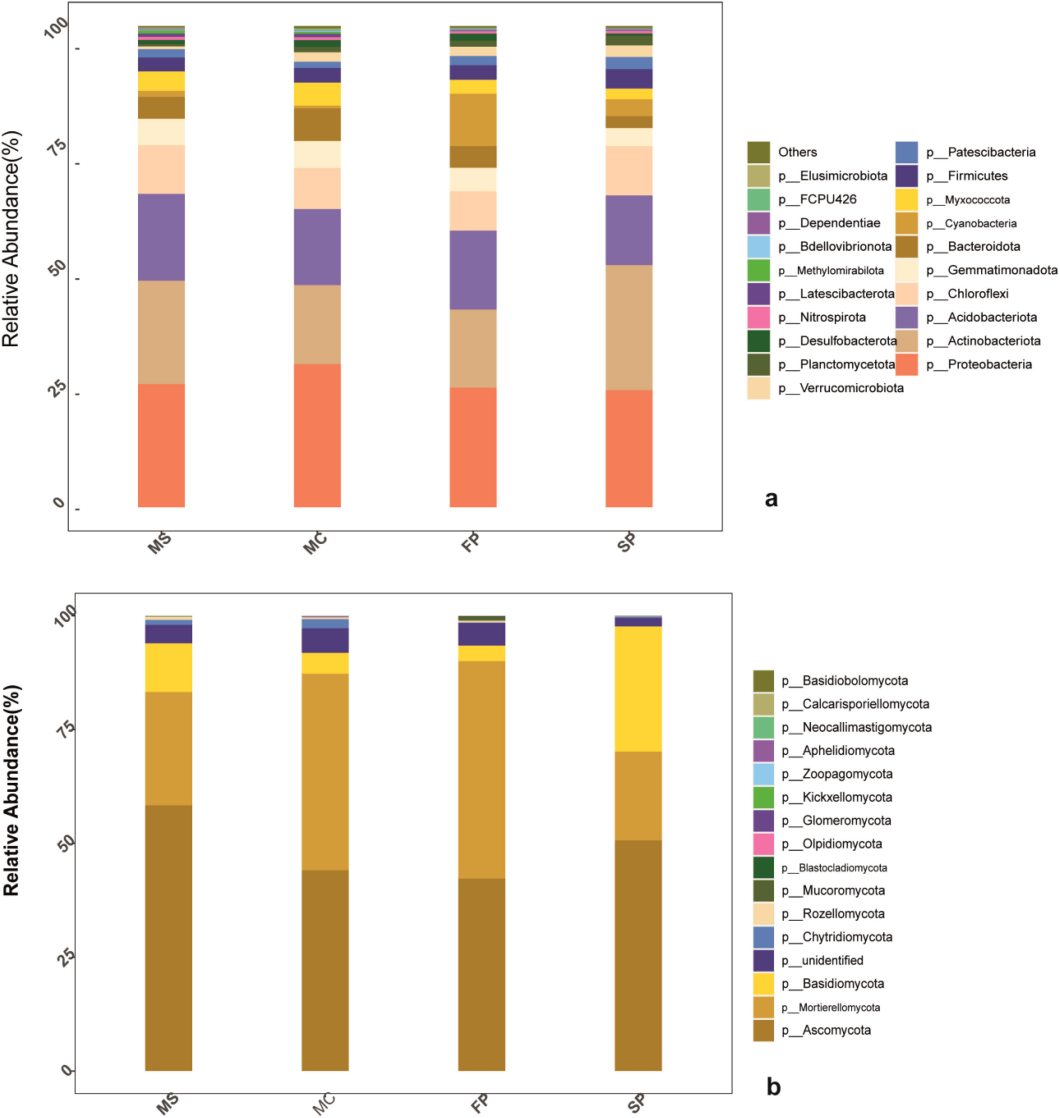
Bar chat **(a)** bacteria taxa **(b)**; fungi taxa. The color scale indicates the relative abundance of each OTU within the samples.

According to metagenomics sequence analysis of bacteria, 2 kingdoms, 38 phyla, 111 classes, 261 orders, 387 families, 677 genera, and 540 species were obtained. In fungi, 2 kingdoms, 17 phyla, 43 classes, 98 orders, 184 families, 316 genera, and 477 species were obtained (**Table 3**). The bacteria composition at phylum level of the top 21 indicated that Proteobacteria, Actinobacteria, Acidobacteria, and Chloroflexi accounted for more than 75% of the relative abundance of the bacteria phyla in MS and SP treatments while in MC and FP accounted more than 60%. Results revealed Proteobacteria to be the most abundant phylum in the entire sample, with a relative abundance of 30% (MC), 25% (MS), 24.6% (FP), and 24.40% (SP). The second most abundant phylum observed was Actinobacteria, with relative abundances of 23%, 17%, 17.4%, and 30% in MC, MS, FP, and SP samples, respectively. The other dominant phyla were Acidobacteria (9.05–13.00%) and Chloroflexi (4.19–6.89%). The phyla classified had a relative abundance of 9.20%, 10.01%, 11.02%, and 6.81% in the (MC), (MS), (FP), and (SP) plots, respectively (**Figure 5a**).

**Table 3:** The number of key taxa of bacteria and fungi community.

Key topology	Bacteria	Fungi
Kingdom	2	2
Phyla	38	17
Classes	111	43
Orders	261	98
Families	387	184
Genera	677	316
Species	540	477

In fungi, phyla, including Ascomycota, Mortierellomycota, and Basidiomycota, accounted for more than 90% of the relative abundance in 90% in FP, 90% in FP, 85% in MC, 80% in MS and 70% in SP. Ascomycota accounted for about 60% of the abundant phyla in the MS plot, followed by SP (49%), MC (42%), and FP (40%). The second abundant phylum was Mortierellomycota, which accounted for about (20%) of the MS plot, followed by the MC (35%), FP (45%), and SP (15%). Meanwhile, Basidiomycota plum was MS (10%), MC (5%), FP (4%) and SP (20%) consecutively (**Figure 5b**).

### 3.4 Taxonomical biomarkers as influenced by soy-corn intercropping

**Figure 6:**
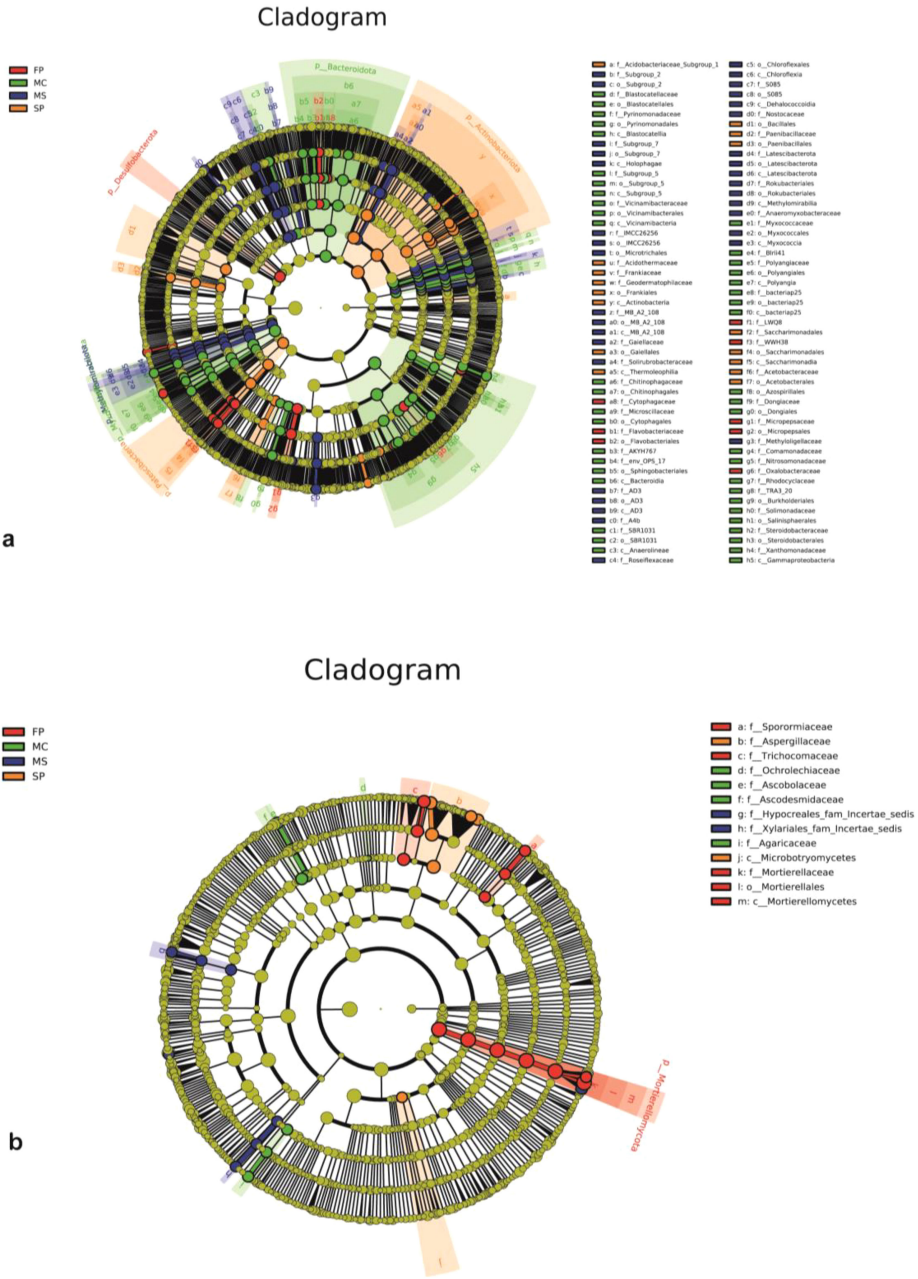
LEfSe cladograms showing the response of **(a)** bacteria and **(b)** fungi genera in rhizosphere soil with different abundance values (LDA score >3; p<0.05).

The LEfSe cladogram was generated to identify distinct bacterial and fungal communities as potential markers of soil microorganisms in MS, MC, FP, and SP soils. LEfSe analysis showed significant variations among 102 bacterial species subjected to the different cropping patterns. We detected 44 taxa in the MC plot, 16 in the SP, 8 in the FP and 34 in the MS. Overall, MC had a significantly higher number of enriched taxa than the rest of the group. Methylomirabilota was enriched in the MS plot, while Bacteroidota and Patescibacteria exhibited the same pattern in the MC and SP, respectively. Moreover, Desulfobacterota and Actinobacteriota were enriched in the FP plot (**Figure 6a**). The LEfSe Cladogram demonstrated that fungi detected in the various cropping systems were divided into13 taxa (**Figure 6b**). The plot FP had a higher number of enriched taxa (5), followed by the MC (4). At the same time, MS and SP were observed to have a low number of taxa (2). Notably, Mortierellomycota was the key enriched fungi phylotype in the FP plot.

The Linear Discriminant Analysis (LDA) estimated the dominant bacteria in the MS, MC, FP, and SP soil samples. In the MS sample, the dominant bacteria were Chloroflexi, Chloroflexales, Roseiflexaceae, Gaiella, and Gaiellaceae. These bacteria were all significantly overrepresented (all LDA scores (log10) > 3) in the MS plot. In the MC group, Gammaproteobacteria, Burkholderiales, Bacteroidia, Bacteroidota, Myxococcota, and Vicinamibacteria were the most abundant microbiota in the control group (LDA scores (log10) > 3). In the FP plot, the dominant microbes observed were Desulfobacterota, Micropepsales, Micropepsaceae, Oxalobacteraceae, and Rhodanobacter. We also observed that Acidothermus, Acidothermaceae, Gaiellales Frankiales, Thermoleophilia, and Actinobacteria were the dominant communities in the SP group and monoculture MS and MC (LDA scores (log10) > 3). Among them, the top 5 distinct genera in each group (P < 0.05, with relative abundance ≥ 0.01%) were selected as key discriminants (**Supplementary Figure 3A**). The distribution of fungi based on LDA scores is shown in (**Supplementary Figure 3B**). In the SP treatment, Aspergillaceae, Penicillium, Aspergillus_flavus, and Microbotryomycetes were more pronounced (all LDA scores (log10) > 3). Additionally, Mortierellaceae, Mortierella, Mortierellomycota, and Westerdykella were considerably overrepresented (all LDA scores (log10) > 3). It was also revealed that Ascobolus, Ascodesmidaceae, Chlorophyllum, and Agaricaceae exhibited similar behavior in the MS plot. Moreover, Mortierella_ambigua and Acrophialophora_levis were significantly overrepresented (all LDA scores (log10) > 3) in the MS.

### 3.5 PICRUSt2 function prediction of soil microbial community function subjected to the soybean-corn intercropping

**Figure 7:**
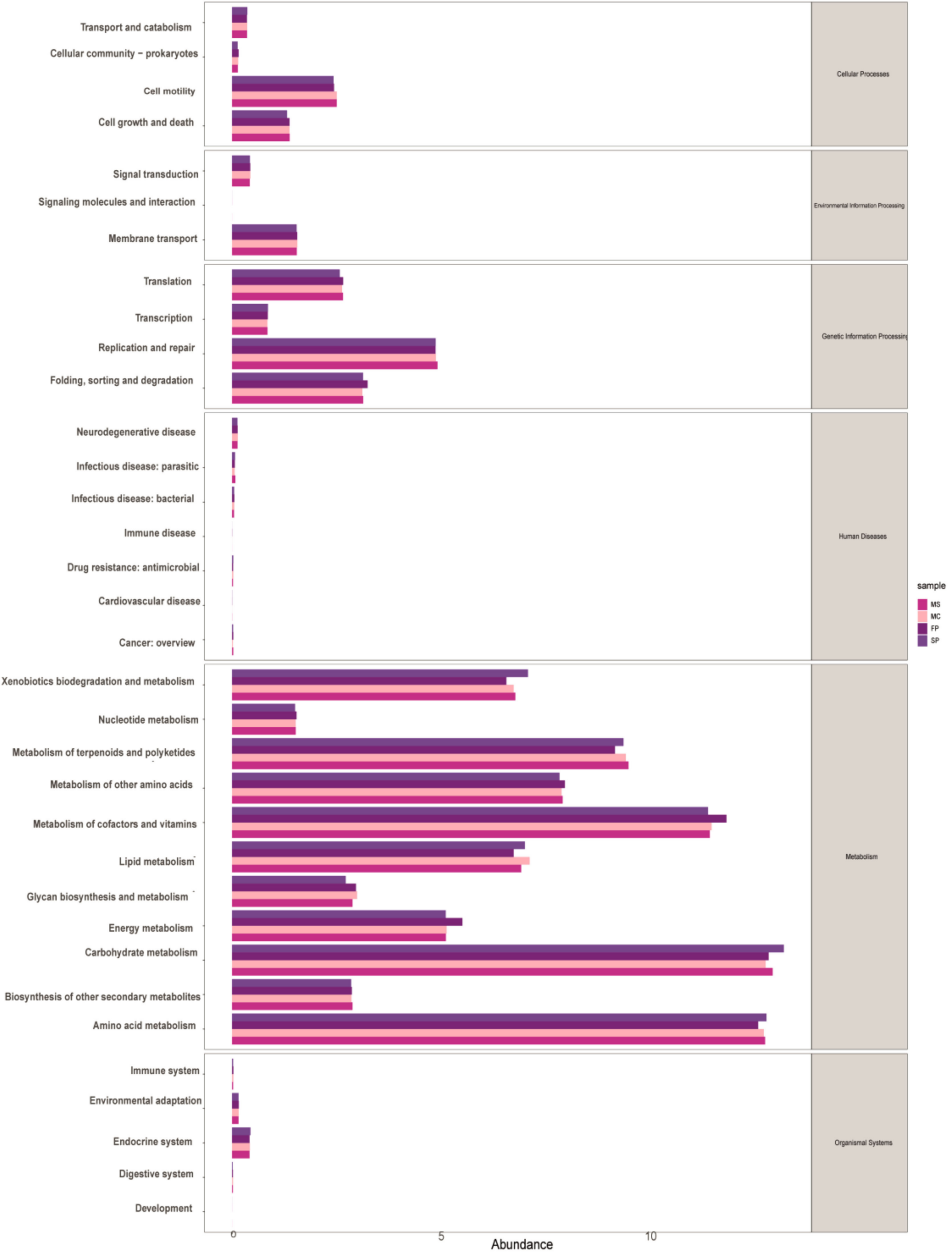
KEGG annotations of PICRUSt2 shows the relative abundance of 34 functional pathways in different cropping patterns.

To investigate the functional differences in the microbial communities along the tiankeng slope, the metagenome was annotated with the metabolic cycles and pathways in the KEGG database. A total of 19789 KEGG orthologues (KO) were annotated in the different cropping patterns. We observed that microbial communities were mainly driven by six functional categories, including metabolism (47%), human diseases (12%), genetic information processing (10%), environmental information processing (9%), cellular processes (9.45%), and organismal systems Biology (3%). The top 5 abundant functional pathways were carbohydrate metabolism, amino acid metabolism, metabolism of cofactors and vitamins, metabolism of terpenoids and polyketides, and xenobiotic biodegradation and metabolism. Additionally, lipid, nucleotide, energy metabolism, and metabolism of other amino acids were also observed. Among the top 11 functional pathways, 3 displayed high relative abundance in SP and 3 exhibited high relative abundance in the FP plot, but no significant difference was observed between MC and SP (**Figure 7**).

The KEGG annotations of PICRUSt2 showed that the microbial communities were also associated with Pentose phosphate pathway, Fatty acid biosynthesis, Steroid biosynthesis, Oxidative phosphorylation, Tetracycline biosynthesis, Cysteine, methionine metabolism, Arginine and proline metabolism, Clavulanic acid biosynthesis, beta-Alanine metabolism, Cyanoamino acid metabolism, Starch and sucrose metabolism, Neomycin, kanamycin and gentamicin biosynthesis, Lipopolysaccharide biosynthesis, Glycolipid metabolism, Inositol phosphate metabolism, C5-Branched dibasic acid metabolism, Carbon fixation pathways in prokaryotes, Carotenoid biosynthesis, and Sulfur metabolism. In addition, the biomarkers of the sample soils were pentose pathway, Glycolysis/Gluconeogenesis, Fatty acid biosynthesis, steroid biosynthesis, and oxidative phosphorylation (**Supplementary Figure 4**).

### 3.6 Agroecosystem microbial community interactions influenced by soybean and corn

The interaction of key soil microorganisms was established using co-occurrence network analysis. The network of bacteria OTUs contained 27 vertices connected by 66 edges, with an average degree of 4.89, a diameter of 4.06 containing, and 0.57 clustering coefficient. The co-occurrence network had an average path length of 2.587. Whereas the network of fungi OTUs contained 27 vertices connected by 63 edges, an average degree of 4.67, a diameter of 4.82, 0.51 clustering coefficient, and an average path length of the network of 2.85 (**Table 4**).

**Table 4:** The table showing microbial co-occurrence networks topological properties.

Network properties	Bacteria	Fungi
Number. edges	66	63
Number. nodes	27	27
Average. degree	4.89	4.67
Number. vertices	27	27
Number. clusters	1	1
Average. Path length	2.59	2.85
Clustering. coefficient	0.57	0.51
Diameter	4.06	4.82
Centralization. betweenness	0.21	0.17
Centralization. degree	0.2	0.17
Edge. connectivity	1	1
Connectance	0.19	0.18

**Figure 8:**
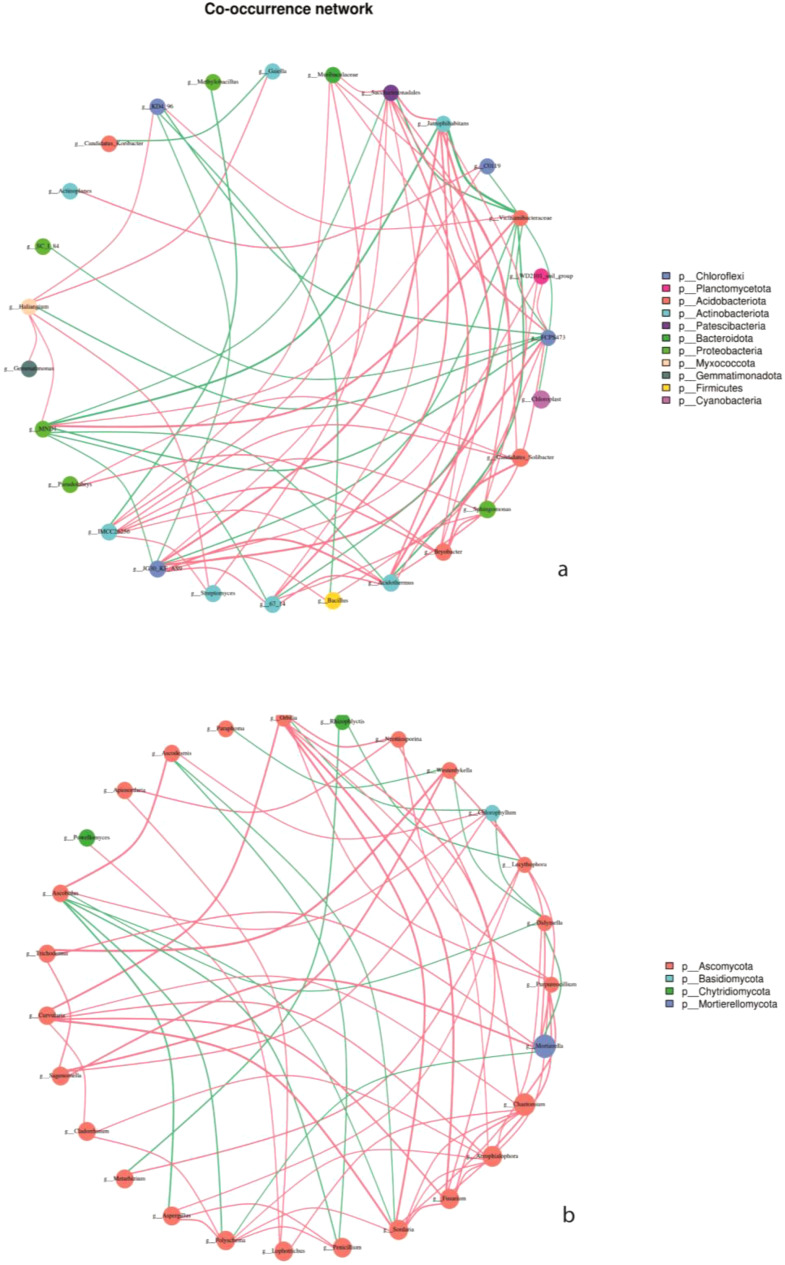
Network interplay diagrams of dominant bacterial **(a)** and fungal **(b)** community; Co-occurrence network, the size of the node represents the size of the abundance, and the thickness of the line represents the size of the correlation. The color of the dots represents the door to which they belong, and the red line shows a positive correlation and green shows a negative correlation.

To perform correlation analysis, the top 30 genus levels of absolute abundance of all samples were selected for analysis. The corresponding gate was used as a legend, and the calculated results were filtered out with P< 0.05 or related value |R|<0.6 (**Figures 8a, b**). The interactions between the dominant 11 bacteria were mainly positive correlations, especially in intercropping systems. We also observed a strong positive correlation among the dominant groups of bacteria, namely Chloroflexi, Actinobacteriota, and Acidobacteriota. Moreover, in fungi communities, the four dominant fungal communities, Ascomycota, were positively connected with Basidiomycota, Chytridiomycota, and Mortierellomycota.

### 3.7 Redundancy analysis revealing microbial composition and soil chemical properties relationship

**Figure 9:**
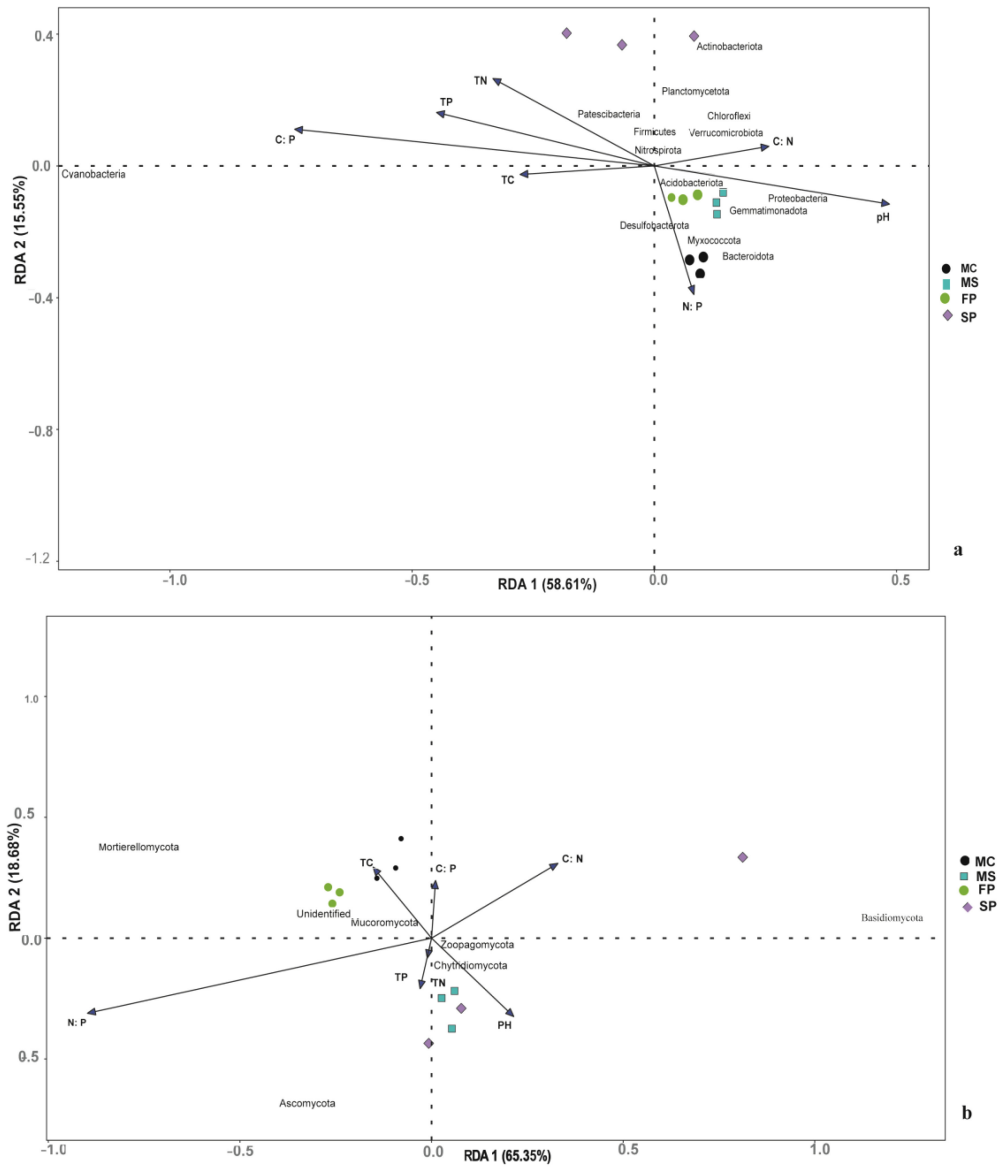
Redundancy analysis (RDA) illustrates the correlation between soil chemical parameters and **(c)** bacterial and **(d)** community distributions. Dots show each sample, while arrows reflect soil chemical characteristics. The length of an arrow represents the degree of correlation between Chemical properties and bacteria community composition.

Redundancy analysis was conducted to assess the relationship between soil microbial composition and chemical properties in the soil samples. Results showed that cropping patterns MS, MC, and FP were closely clustered. The intercropping pattern SP was observed to be distantly clustered. This indicates that soil bacterial composition was significantly affected by cropping patterns. The RDA1 and RDA2 explain 76.32%, and 17.32% of the total variance in the bacterial community, respectively (**Figure 9a**). Based on our research, we found out Proteobacteria positively correlated with salinity concentration, EC and pH in the MC pattern, which according to observation had a notable significant concentration of salt, EC and pH. Meanwhile, there was no correlation between Firmicutes and soil properties in our study. We also found that Chloroflexi had a high relative abundance in MS and SP and was positively correlated with soil SOM and TC. Furthermore, soil chemical properties significantly influenced Actinobacteria, and it had a higher relative abundance of SP patterns. Meanwhile, Cyanobacteria was negatively correlated with most soil chemical properties.

Fungi RDA analysis indicated that RDA1 and RDA2 accounted for 68.97% and 21.03% of the total variance in the fungal community (**Figure 9B**). Moreover, results indicated that Basidiomycota, Glomeroimycota, Rozellomycota, Blasiocladiomycota, Mucoromycota, and Mortierellomycota in fungi phyla were significantly correlated with soil chemical properties. Ascomycota was negatively correlated with most soil chemical factors except EC, which was more abundant in MS and SP. This explains that soil pH, Salt content, EC, TOC, TN, TP, TC and SOM were the significant drivers of the community composition shift in the four intercropping patterns.

## 4 Discussion

### 4.1 Soybean-corn intercropping enhancing the changes in soil chemical properties, elemental ratios and salt concentration

The intercropping of soybean and corn is observed to be among the best practical approach of improving the soil properties (Kamara et al., 2019). In our study, it was observed that soil TN, TP, TC and SOM were significantly lower in monoculture patterns MC and MS compared to intercropping patterns FP and SP at (p<0.05) (**Figures 2a–d**). These findings are in agreement with studies reported that intercropping of soybean and corn can improve soil SOM, TC, TN, and TP (Tang et al., 2021; Duchene et al., 2020). Soil chemical properties are essential to soil nutrients crucial to soil circulation of nutrients in the agroecosystem (Sun et al., 2009). In addition, our study found that soybean and corn intercropping influenced the key ecosystems C, N, and P elements, hence influencing C: N, C: P, and N: P chemical element ratios (**Figure 2**) which was also reported by (Liu et al., 2016; Ma et al., 2023). The ratio of soil C: N is a primary component of soil organic matter and the higher values of these ratios indicate that soil is higher in microbial activities and of good quality (Chen and Chen, 2021).

Furthermore, soil pH was significantly lower in the FP and SP plots compared to monoculture MC and MS. This result suggested that soil intercropping of soybean and corn effectively regulates soil pH in agroecosystems (Chen et al., 2019). The change in soil pH might be caused by organic acids secretions in intercropping groups (Li et al., 2022). The salinity content was significantly reduced in FP and SP compared to monoculture patterns, indicating that the combination of plants in intercropping patterns significantly lowered salt concentration compared to monoculture patterns (Ahmad, 2011).

### 4.2 Changes in soil community composition and diversity in agroecosystem

Our results indicated that the diversity indices Chao 1 and Shannon of bacteria were observed to be low in the intercropping pattern. However, the diversity indices of the fungi community were higher in the FP and SP plots but lower in the MS and MC (**Figures 4a, b**). Corresponding to our results, the intercropping of soybean-corn influences the diversity structure of soil fungi (Sugiyama, 2019). In intercropping, soybeans host rhizospheric microbes in their root nodules, which are essential in soil biological processes such as nitrogen fixation, and phosphorus solubilization hence affecting the diversity of soil microbes in the soil (Jacoby et al., 2017). Consistent with previous studies microbial community diversity is affected by the plant type and planting patterns that influence the below-ground plant interactions (Zhou et al., 2011). Plant type and cropping systems alter the microbial community structure and diversity by releasing root secretions, which are important carbon sources in rebuilding soil microbes (Wu and Yu, 2019).

The bacterial and fungi community composition structure differed significantly between cropping patterns. Proteobacteria was reported to be the most abundant phylum in the entire sample because of their nature and habitats high abundance was reported in MC, followed by MS, corresponding to the findings reported by (Yang et al., 2020). Actinobacteria was the second most abundant bacteria found to be high in the MS and SP but low in the MC and FP treatments (**Figure 5a**). According to the previous results, it has been reported to be an ecosystem remediation agent, indicating the possibility of high remediation processes in the MS and SP patterns (Mawang et al., 2021). In fungi, Ascomycota had a significantly high relative abundance in MS (60%) (**Figure 5b**). The abundance of Ascomycota increased in the MS treatment is due to nitrogen concentration present due to nitrogen-fixing ability and rhizodeposition, which is important for fungi growth in rhizosphere soil (Kalu et al., 2021). In this study, soybean-corn intercropping was observed to have a significant effect on the abundance and distribution of many soil microbes primarily due to the ability of soybean to regulate microbial communities by modifying soil chemistry, which is an important determinant of soil microbial properties (Tang et al., 2021).

### 4.3 Changes of soil community biomarkers and predicted functions due to soybean-corn intercropping

Studying soil microbial communities helps us to predict the functions of soil important biomarkers and their influence on agroecosystem’s function (Hu et al., 2018). This study revealed substantial variations among 102 bacterial and 13 fungi taxa in the LEfSe of the four cropping patterns. The soil microbes were more sensitive to soybean-corn intercropping, and the quantity of enriched microbial taxa became less pronounced after the MC>MS>SP>FP in bacteria and FP>MC> MS>SP in fungi. This indicates that cropping patterns significantly influenced soil microbial variations (Duchene et al., 2020). In bacteria, Methylomirabilota, Bacteroidota, and Patescibacteria enriched considerably in the MS, MC and SP plots, respectively. Moreover, Desulfobacterota and Actinobacteriota exhibited a similar phenomenon in the FP plot (**Figure 6a**). In fungi, Mortierellomycota was the most enriched fungi in the FP treatment. This shows that soil microbes are highly sensitive to intercropping patterns (Sun et al., 2009). The soil microbial biomarkers are affected by chemical compounds such as flavonoids and energy released by plant roots of soybean and corn (Bais et al., 2006; Hu et al., 2018). Microbial analysis in this study indicated that the microorganisms are involved in pollutant degradation, nitrogen fixation, and carbon fixation. Studies have documented that the soybean and corn intercropping patterns significantly influence the predicted functions of soil microbes (Li et al., 2022; Zhang et al., 2023).

### 4.4 Correlation of microbial community and soil chemical properties under different intercropping patterns

In studying the influence of soil properties on microbial community composition, it is important to understand the interactions of microbial communities in a particular environment. The co-occurrence networks provide insight into understanding the interaction, variations and functions of soil microbes in agroecosystem (Wu et al., 2023). In this study, the bacteria network had more edges and diversity than the fungal network (**Table 3**). Study indicated that the interaction of soil microbial communities contributes to the formation of a complex network of microorganisms in the soil which is important for soil biological processes (Dong et al., 2022). This study discloses that the formation of bacteria and fungi complexes is associated with the nature of the environment in which microbes are found (Schmidt and Waldron, 2015) and suggests that most bacteria and fungi had similar functions and were mutually beneficial to each other (**Figure 9**).

Studies indicated that the interaction of soil chemical properties and soil microbes is important in nutrients, energy transfer at different trophic levels and health of coastal agroecosystem (Ambrosini et al., 2016). RDA analysis indicated that the dominant bacterial and fungal communities were significantly correlated with salinity content, EC, pH, TP, TN, TC, SOM, TOC and soil chemical element ratios factors which influenced unique bacterial and fungal communities in coastal agroecosystems (Ladau and Eloe-fadrosh, 2019). Supporting our study, the salinity content influenced the composition of soil microbial communities (Xu et al., 2020).

Based on our RDA results of bacteria and soil chemical analysis presented in (**Figure 9A**) we found out Proteobacteria was correlated with salt content, EC and pH. Also, no significant correlation between Firmicutes and soil properties was observed, meanwhile Firmicutes are known to be responsible for nitrogen fixation and growth promotion (Lin et al., 2019) and are widely found in saline soil (Xu et al., 2020). We also found that Chloroflexi had high relative abundance in MS and SP and was positively correlated with soil SOM, and TC. Corresponding to our results Chloroflexi is vital in the conversion of C to soil organic carbon, nitrate, nitrite, and ferric iron (Speirs et al., 2019; Spieck et al., 2020).

Furthermore, Actinobacteria was significantly influenced by soil chemical properties and had higher relative abundance in SP patterns. According to the previous study, Actinobacteria is one of the common phyla in ecosystems and is found in soil with high pH (Polti et al., 2014). They are ecologically important due to their ability to produce secondary metabolites important for the circulation of nutrients and degradation of soil contaminants such as hydrocarbons, pesticides, and heavy metals (Mawang et al., 2021).

RDA results suggested that Basidiomycota, Ascomycota and Mortierellomycota among fungal phyla which were substantially linked with soil nutrients (**Figure 9b**). Basidiomycota was positively correlated with all chemical properties except EC and had high relative abundance in SP. Ascomycota correlated with EC and observed to have a high relative abundance in SP and MS. Basidiomycota and Ascomycota are important fungi in ecosystem decomposition activities (Praeg et al., 2019; Delgado et al., 2021). In this study, Mortierellomycota was positively correlated with all chemical properties except EC and the relative abundances were high in MC and FP. Previous research suggested Mortierellomycota can transform soil phosphorus into usable form which can be taken by plants hence productivity of the soil (Wu et al., 2019).

## 5 Conclusion

Our results revealed the soybean and corn intercropping patterns have significant effects on the diversity, composition and functional characteristics of the soil microbial communities. The soybean and corn intercropping not only affected microbial community interactions in the coastal saline agroecosystem but also affected the soil’s chemical properties and their proportions. Our study provides a new insight into the significance of soybean and corn intercropping in improving microbial interactions and their functions in coastal agroecosystems. Further research using different soybean and corn varieties and patterns is required to validate these findings.

## Data Availability

The datasets presented in this study can be found in online repositories. The names of the repository/repositories and accession number(s) can be found in: https://www.ncbi.nlm.nih.gov/bioproject/PRJNA1224044.
