# Supplementary material for: Exploring the change of coastal saline agroecosystem diversity, composition and predicted function of soil microbial community mediated by soybean and corn intercropping
Source: Front Plant Sci. 2025 Jun 26;16:1427196. doi: 10.3389/fpls.2025.1427196 (PMC12240985; doi:10.3389/fpls.2025.1427196)
Supplement: Supplementary file 1 [file Image1.pdf]

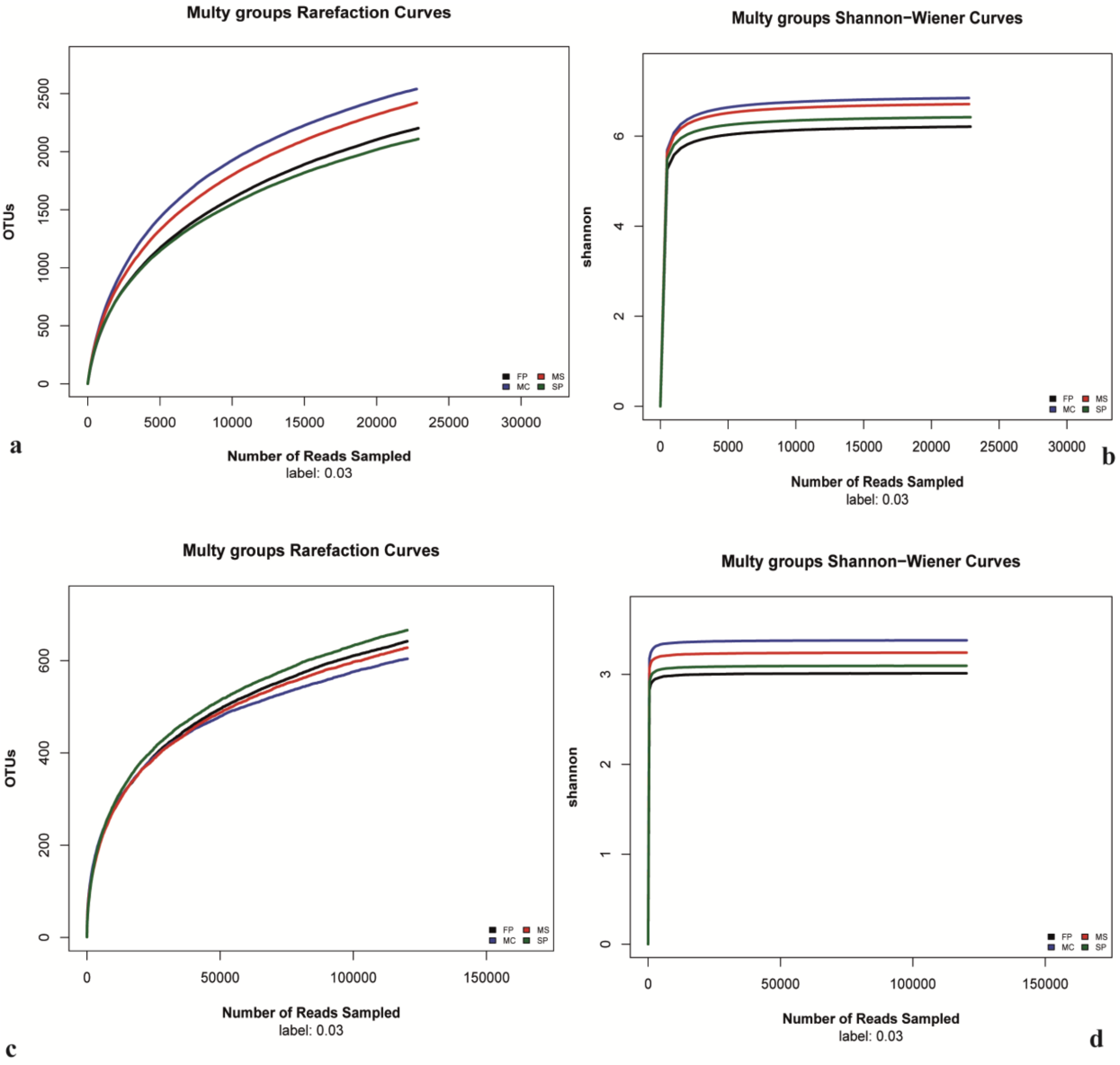

Supplementary figure 1. Multy group Rarefaction curve (A) Shannon-Wiener index of (B) of bacteria. Multy group Rarefaction curve (C) Shannon-Wiener index of (D) of fungi.

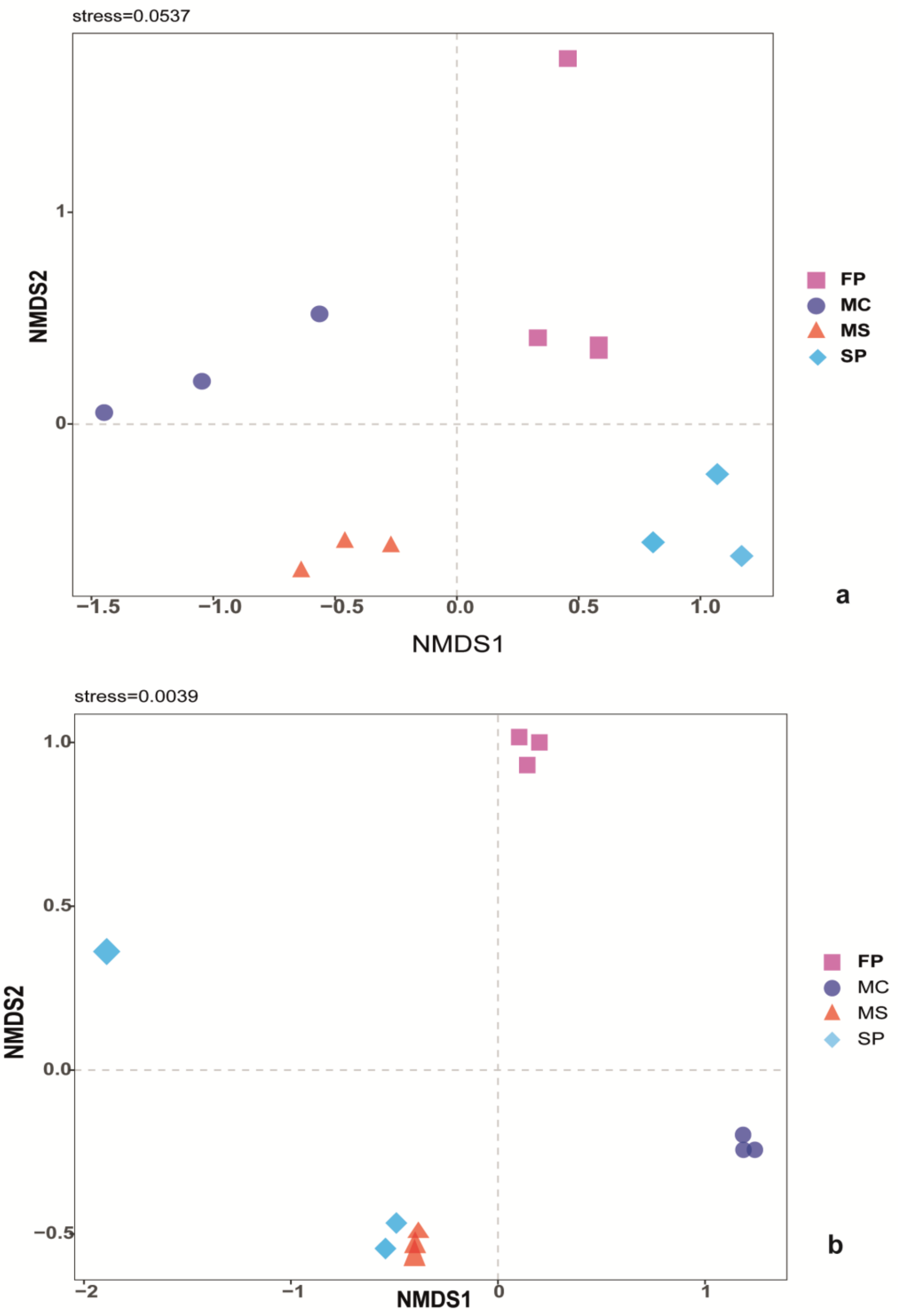

Supplementary figure 2. The NMDS of bacteria (A) and fungi (B) community for different intercropping patterns.

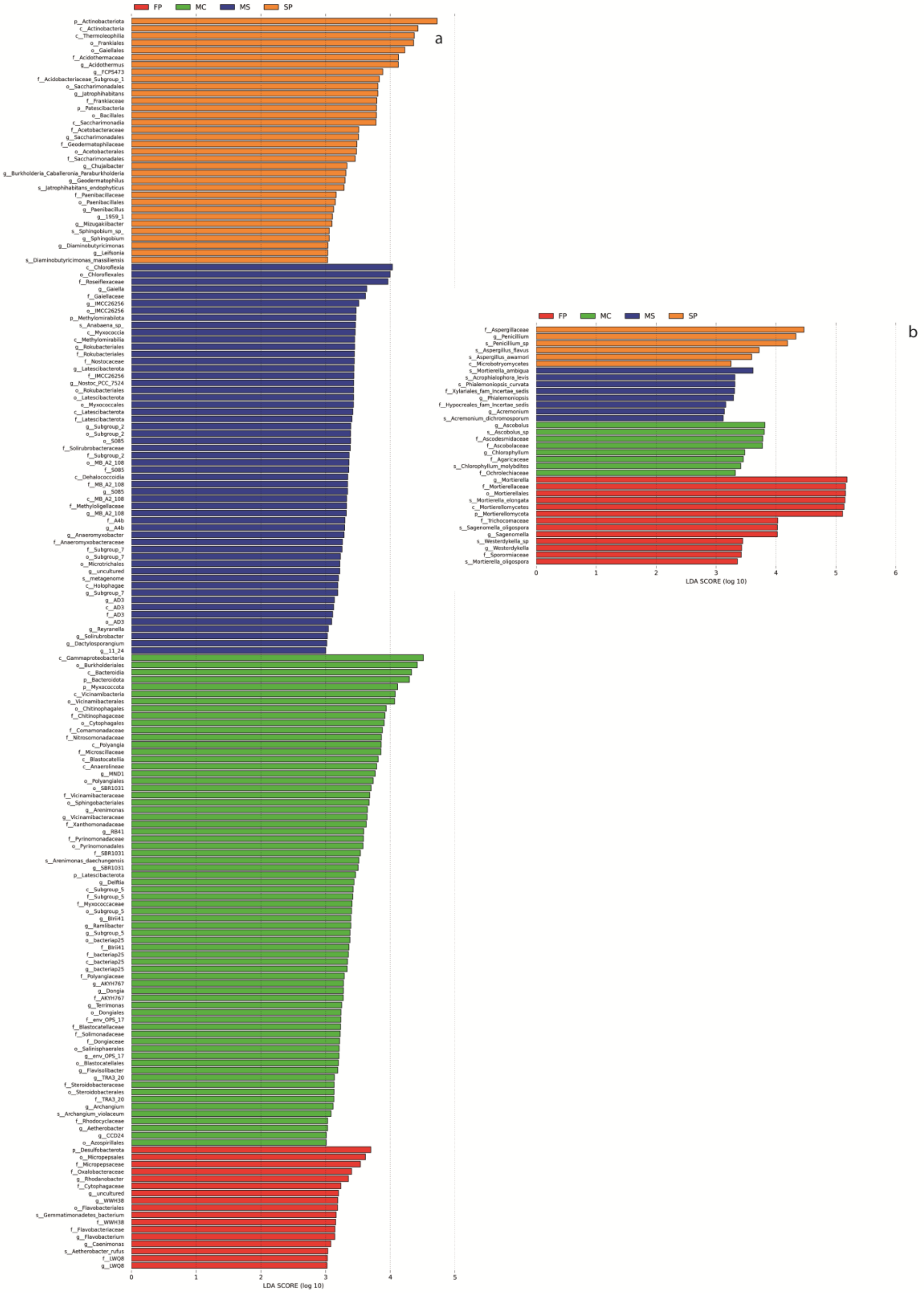

Supplementary Figure 3. Distribution Histogram based on Linear discriminant analysis (LDA) effect size (LEfSe) indicating enriched bacteria (A) and fungi (B) genera associated with MS (blue), MC (green), FP (red) and SP (yellow).

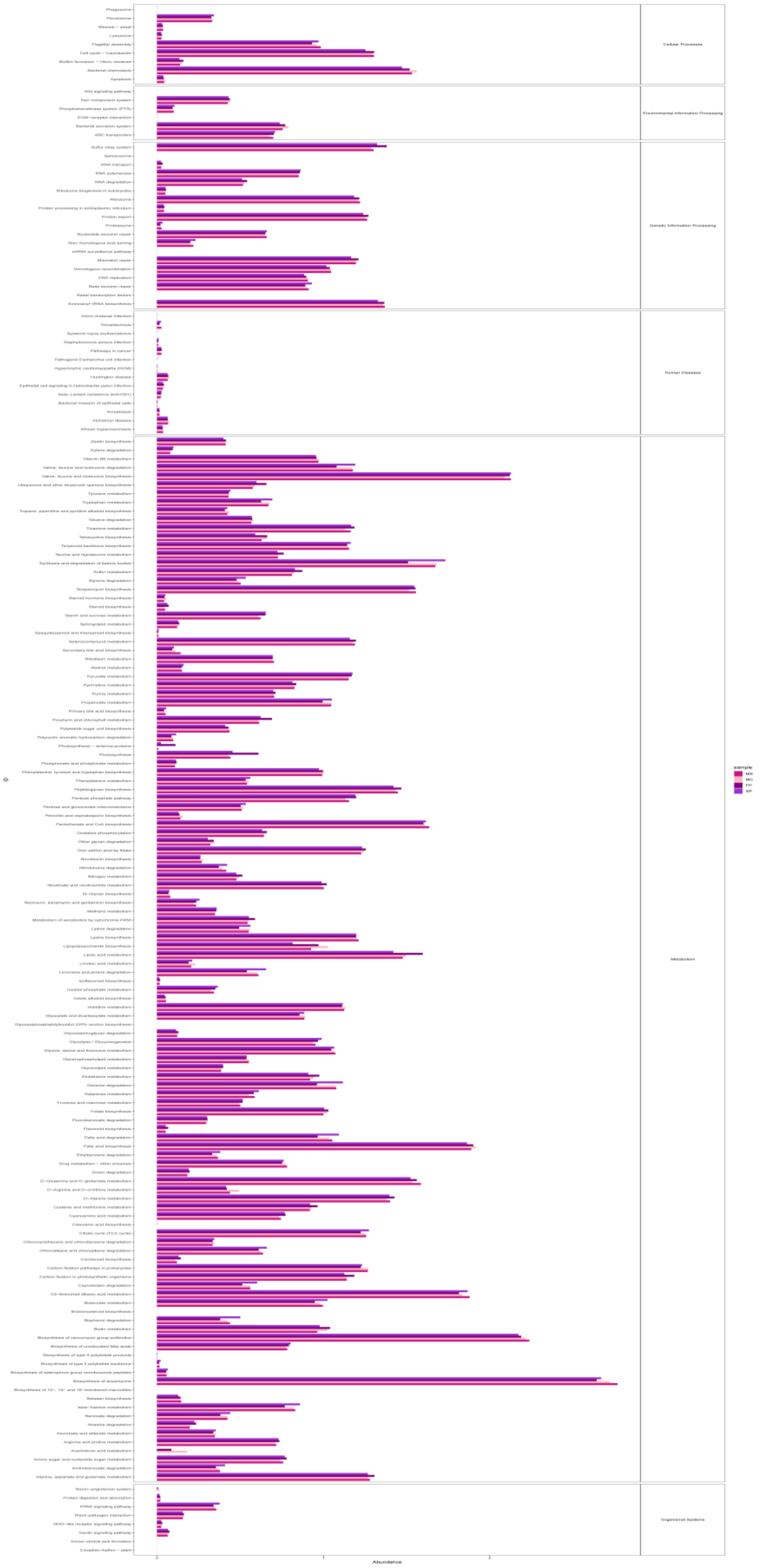

Supplementary figure. 4. KEGG annotations of PICRUSt2 show the relative abundance of 34 functional pathways in different cropping patterns.
